# Regenerative Effect of a ROCK Inhibitor, Y-27632, on Excitotoxic Trauma in an Organotypic Culture of the Cochlea

**DOI:** 10.3389/fncel.2020.572434

**Published:** 2020-11-17

**Authors:** Yutaka Koizumi, Tsukasa Ito, Kunio Mizutari, Seiji Kakehata

**Affiliations:** ^1^Department of Otolaryngology-Head and Neck Surgery, Faculty of Medicine, Yamagata University, Yamagata, Japan; ^2^Department of Otolaryngology-Head and Neck Surgery, National Defense Medical College, Saitama, Japan

**Keywords:** cochlea, spiral ganglion, Y-27632, hearing loss, inner ear, synapse, regeneration, Rho-associated coiled-coil containing protein kinase

## Abstract

In the past, most inner ear diseases were thought to start with the impairment of the sensory epithelium of the cochlea before subsequently progressing to secondary neural degeneration. However, recent studies show that loss of primary synapses accompanied by excitotoxic degeneration of peripheral axons is likely to be the underlying pathology in sensorineural hearing loss. Rho-associated coiled-coil containing protein kinase (ROCK) inhibition has been reported to have neuroprotective and regenerative effects on synaptic pathways. Therefore, we analyzed the effect of ROCK inhibition using Y-27632 in a model of peripheral axonal damage in the spiral ganglion neurons created using the glutamate agonists, *N*-methyl-D-aspartate (NMDA) and kainic acid, to induce excitotoxic trauma in the explanted cochlea. The number of axons projecting to hair cells in the cochlea treated with Y-27632 was significantly greater than those in the cochlea treated only with NMDA + kainic acid. Furthermore, there was a significant increase in synapses between the spiral ganglion and the inner hair cells in the cochlea treated with Y-27632. The findings of this study suggest that ROCK inhibition could be a potential strategy for the regeneration of peripheral axons in the spiral ganglion and synapse formation in the inner hair cells of a cochlea that has sustained excitotoxic injury, which is one of the primary etiologies of inner ear disease.

## Introduction

The etiology of sensorineural hearing loss (SNHL) involves two main mechanisms, the loss of sensory epithelium and/or the loss of sensory neurons (Wang et al., 2002; Sugawara et al., 2005; Liberman and Kujawa, 2017). The sensory epithelium, which includes hair cells and supporting cells, is the primary receptor of sound. Spiral ganglion neurons (SGNs) form synaptic connections with hair cells, which receive sound signals, convert them into action potentials, and convey them to the auditory center via SGNs. The conventional thinking has been that inner ear disease begins with impairment of the sensory epithelium of the cochlea and is followed by secondary neural degeneration (Johnsson, 1974; Bohne and Harding, 2000). Therefore, primary neural degeneration of the cochlea without loss of hair cells is considered to be a rare cause of SNHL. However, recent studies have shown that primary synapse degeneration, known as cochlear neuropathy, which causes a reduction in the amplitude of wave I in the auditory brainstem response (ABR) without elevation of the ABR threshold caused by loss of the synaptic ribbon (Kujawa and Liberman, 2009, 2015), is a common underlying pathology in SNHL. This type of hearing dysfunction has recently been described as hidden hearing loss, the pathology of which is strongly associated with the pathogenesis of tinnitus (Schaette and McAlpine, 2011; Schaette et al., 2012) and hyperacusis (Hickox and Liberman, 2014). The above-mentioned lesions are now recognized as a potential therapeutic target for SNHL.

Rho-associated coiled-coil containing protein kinase (ROCK) is a serine-threonine protein kinase that has been identified as a target protein of small molecular weight GTP-binding protein Ras homologous (Rho) (Madaule and Axel, 1985). Similar to the Rho family, low molecular weight G protein Ras-related C3 botulinum toxin substrate (Rac), and cell division cycle 42 (Cdc42) (Didsbury et al., 1989; Munemitsu et al., 1990), Rho has a specific action in the remodeling of actin (Hall, 1998). ROCK inhibitors inhibit the Rho/ROCK pathway and have multiple effects, including increased blood flow, neuroprotective actions, and neurosynaptic regeneration, and are used clinically as therapeutic agents for glaucoma and cerebral vasospasm. Several clinical trials have been conducted in cardiovascular disease (Shimokawa et al., 2002; Fukumoto et al., 2005; Vicari et al., 2005). Furthermore, ROCK inhibitors have been reported to have a beneficial effect on axonal regeneration in the central nervous system (Sarner et al., 2000; Yamaguchi et al., 2001). There have also been reports on the effect of promoting nerve fiber elongation by using a ROCK inhibitor in the cochlear nerve and in the central nervous system (Lie et al., 2010; Defourny et al., 2013). Therefore, we hypothesized that ROCK inhibitors would affect remodeling of the synapses between hair cells and axons in the SGN even after the cochlea has been damaged by an inner ear disorder.

To validate this hypothesis, we examined the effects of ROCK inhibition on damaged auditory nerve fibers in an organotypic culture of the cochlea. The most straightforward method to test the effectiveness of candidate compounds would be to perform *in vitro* research using the cultured organ. However, there is no established cochlear synaptopathy *in vitro* model. Therefore, we used a *N*-methyl-D-aspartate (NMDA) and kainite-induced auditory nerve damage culture model in our study. Brief exposure to NMDA and kainite (NK) was used to induce the auditory nerve damage. The exposure led to the loss of the synapses between the inner hair cells (IHCs) and synapses in the SGN as well as degeneration of the distal type 1 peripheral axons in the SGN, thus mimicking, *in vivo*, the damage caused by excitotoxicity or noise (Wang and Green, 2011). In addition, this excitotoxicity model has been hypothesized to represent critical aspects of neuropathy or synaptopathy (Han et al., 2019).

## Materials and Methods

C57BL/6J mice were sourced from Japan SLC, Inc. (Hamamatsu, Japan) on postnatal day 4, 5, or 6. A total of 29 mice, and 58 cochleae were employed in this study. The mice were placed on ice to induce hypothermic anesthesia and euthanized by decapitation. The inner ear was harvested from the mouse temporal bone and the cochlear tissue was isolated in phosphate-buffered saline (PBS) under a stereomicroscope.

The study was approved by the Yamagata University Animal Experiment Committee (approval number, 28156) and carried out in accordance with the rules for animal experimentation at Yamagata University.

### Organotypic Culture of the Cochlea

The dissected cochlear tissue isolated from mice of either sex was cultured in Hanks solution. To obtain a flat cochlear surface preparation, the spiral ganglia, Reissner’s membrane, and most of the basal cochlear segment were removed. The explants were plated onto 4-well plates coated with 0.01% poly-L-ornithine (Sigma, St. Louis, MO, United States) and 50 μg/ml laminin (BD Biosciences, Franklin Lakes, NJ, United States). All cultures were maintained in a 5% CO_2_/20% O_2_-humidified incubator. The composition of the culture medium was as follows: Dulbecco’s modified Eagle’s medium/Ham’s F12 medium (Gibco Life Technologies, Paisley, United Kingdom), 10% heat-inactivated fetal bovine serum, 25 mM HEPES (Gibco), N-2 Supplement (Gibco), B-27 Supplement (Gibco), and 100 U/l penicillin G (Wako, Osaka, Japan). The organotypic culture was maintained for up to 72 or 96 h at 37°C.

### Excitotoxic Injury of Cochlear Tissue in Organotypic Culture and Treatment With a ROCK Inhibitor

Damaged cochlear tissue were created as described elsewhere (Wang and Green, 2011). Following the original method, *N*-methyl-D-aspartic acid 0.5 mM (NMDA; Tocris Bioscience, Ellisville, MO, United States) 0.5 mM and kainic acid (Tocris Bioscience; NK treatment) were reacted in a culture solution for 2 h.

The following three groups were compared to investigate the effects of a ROCK inhibitor, Y-27632 (257-00511, Wako) in a model of excitotoxic injury of cochlear tissue: a control group; an NK group (NMDA 0.5 mM + kainic acid 0.5 mM for 2 h, followed by washing and culture in normal culture medium); and a ROCK inhibition group (washed after treatment with NK with addition of 10 μM Y-27632 in normal culture medium). Immunohistochemical evaluations were performed at 24 and 72 h after culture.

### Quantitative RT-PCR

Before and after NK treatment, harvested cochleae tissues were collected and stored in RNAlater (Ambion, Austin, TX, United States) (*n* = 6 per group). Total RNAs were extracted with an RNeasy Mini Kit (QIAGEN, Valencia, CA, United States) according to the manufacturer’s instructions. Quantitative RT-PCR was performed on a Thermal Cycler Dice Real Time System using the One Step SYBR PrimeScript PLUS RT-PCR Kit (RR096A; TaKaRa Bio, Shiga, Japan). Forward (F) and reverse (R) primer sequences were RhoA-F, 5′-AGCTTGTGGTAAGACATGCTTG-3′ and RhoA-R, 5′-GTGTCCCATAAAGCCAACTCTAC-3′, ROCK1-F, 5′-GACTGGGGACAGTTTTGAGAC-3′ and ROCK1-R, 5′-GGGCATCCAATCCATCCAGC-3′ and ROCK2-F, 5′-TTGGTTCGTCATAAGGCATCAC-3′ and ROCK2-R, 5′-TGTTGGCAAAGGCCATAATATCT-3′. PCR cycling conditions included 40 cycles of 95°C for 5 s and 60°C for 30 s. Expression was determined using the ΔΔCt method with glyceraldehyde 3-phosphate dehydrogenase (GAPDH) as internal control. All reactions were performed in duplicate. mRNA expression was reported as n-fold GAPDH mRNA. For assessment of relative mRNA expression, levels were standardized to cochlear samples extracted before NK treatment.

### Immunohistochemistry

The whole-mounted cochlear tissues were reacted with 4% paraformaldehyde/PBS at room temperature for 1-h for fixation. After washing with PBS, 0.3% Triton X, and 5% BSA/PBS, the specimens were reacted at room temperature for 1-h for blocking and permeabilization. The following primary antibodies were reacted at 4°C for 16 h: Rabbit anti-ROCK1 (1:100 Abcam, Cambridge, United Kingdom: ab134181); Rabbit anti-ROCK2 (1:100 Abcam, Cambridge, United Kingdom: ab125025); Rabbit anti-NF200 (1:1000 Sigma: N4142), Chicken anti-NF 200 (1:1000 Chemicon, Temecula, CA, United States: MAB5262), Rabbit anti-NF200 as an auditory nerve marker, Rabbit anti-Myo7a (1:500, Proteus Biosciences Inc., Ramona, CA, United States: 25-6790) as a hair cell marker, Mouse (IgG1) anti-CtBP2 (1:1000 BD Biosciences, San Jose, CA, United States: 612044) as a presynaptic marker, Mouse (IgG2a) anti-PSD95 (1: 5000 Abcam: ab 2723) as a postsynaptic marker. After washing with PBS, the following secondary antibodies, diluted 500-fold, were reacted at 4°C for 16 h: Alexa 405 Goat anti-Rabbit IgG (Invitrogen, Carlsbad, CA, United States: A31556), Alexa 488 Donkey anti-Rabbit IgG (Invitrogen: A21206), Alexa 488 Goat anti-Chicken IgG (Invitrogen: A11039), Alexa 488 Goat anti-Mouse IgG2a (Invitrogen: A 2131), and Alexa 568 Goat anti-Mouse IgG1 (Invitrogen: A-21124). After washing with PBS, the samples were mounted with glycerol and observed.

### Quantitative Analysis of Peripheral Axons in the SGN and Synapses

The fluorescently labeled samples, including SGN somata and axons, IHCs, synapses, and other structures, were observed using an LSM-700 confocal laser microscope (Carl Zeiss Microscopy GmbH, Jena, Germany). Optical sections in the x–y plane (z-sections) were recorded at 0.5-μm intervals in the z-axis. The resulting confocal image series (z-stack) contained a three-dimensional image in the entire volume of the explant. The z-stack was reconstructed (to view a plane perpendicular to the x–y plane) as necessary using ImageJ^1^ or Photoshop CC (Adobe, San Jose, CA, United States).

We analyzed the number of peripheral axons in the SGN projecting into the IHCs using z-stack images. The methods used to quantify the peripheral neurons, hair cells, presynaptic ribbons, and postsynaptic density in the SGN were mostly consistent with a previous report (Wang and Green, 2011). Every IHC, outer hair cell (OHC), NF200-labeled axon, CtBP2 punctum, and PSD-95 punctum was counted within three randomly chosen volumes in the organ of Corti in the middle turn of the cochlea. Given that the number of afferent synapses on each IHC varies according to location, with a greater number in the middle than in the apex or base (Meyer et al., 2009), we restricted our study to the middle of the cochlea to minimize any location-dependent effects as found in previous research (Wang and Green, 2011).

**FIGURE 1:**
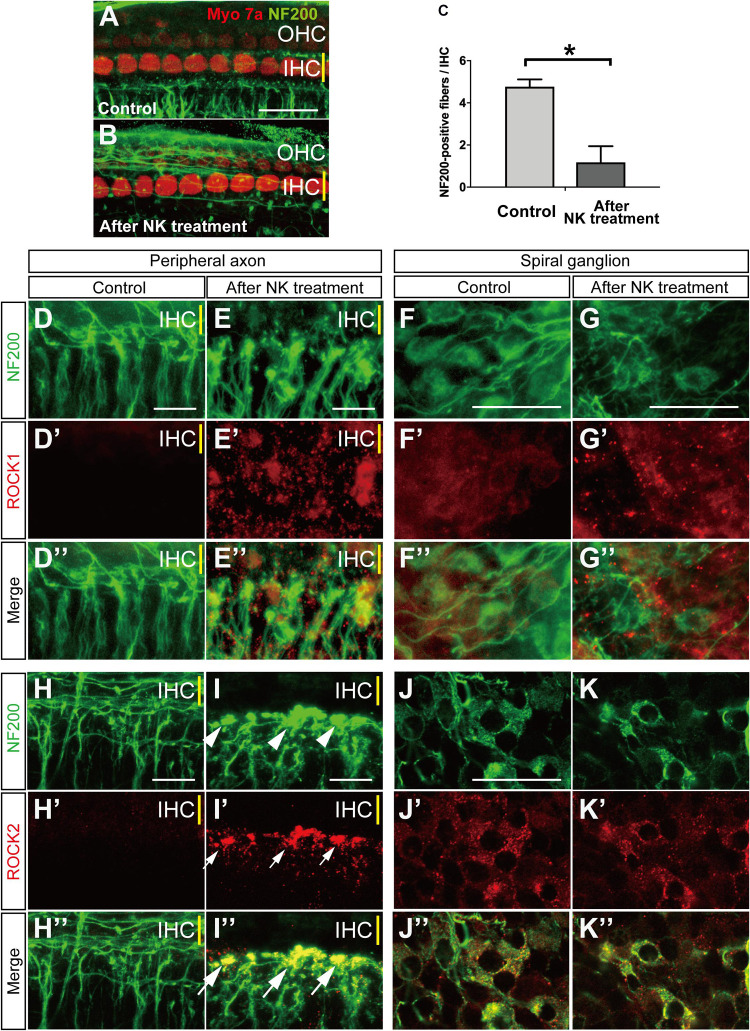
Morphological changes and ROCK1, ROCK2 expression with and without excitotoxic damage caused by NMDA/kainate (NK) treatment in an organotypic culture of the organ of Corti. **(A,B)** Organotypic cochlear explants prepared from the middle turn of the neonatal mouse and cultured hair cells are labeled with anti-myosin 7a (red) and spiral ganglion peripheral axons with anti-NF200 (green). **(A)** A projection image of a confocal series through an explant, including the inner hair cell (IHC) row (yellow line) and the three outer hair cell (OHC) rows, showing the characteristic innervation pattern. **(B)** The peripheral axons of the spiral ganglion showed significant degeneration after 2 h of treatment with NK. **(C)** Peripheral axon counts immediately after 2 h of treatment with NK or no NK (controls) (*n* = 6). ^∗^Indicates a significant difference (*p* < 0.05). The data are shown as the mean ± standard deviation. The projection images of a confocal series of cultured explants stained by anti-NF200 antibody [green, **(D–K)**], anti-ROCK1 antibody [red, **(D′–G′)**] and merge images **(D″–G″)**, anti-ROCK2 antibody [red, **(H′–K′)**], and merge images **(H″–K″)**. **(H,I)** SGN peripheral processes, which are the radial fibers that innervate the IHC row, were markedly degenerated and formed debris (white arrowheads) at the ending of the processes **(E,I)**. After excitotoxic damage by NK, ROCK1 expression was slightly elevated around the fibers **(E′,E″)** and the spiral ganglions **(G′,G″)**. At the sites of debris formation, ROCK2 expression was significantly elevated consistent with the debris [white arrows in panel **(I′,I″)**]. However, there were no changes in the ROCK2 expression pattern in the spiral ganglion cell body **(J′,K′)**. Scale bar: 20 μm.

**FIGURE 2:**
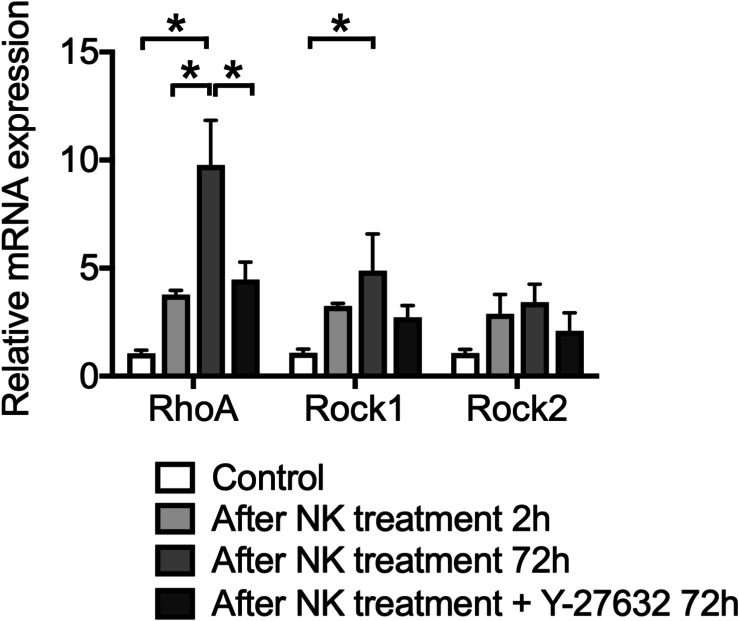
The effect of Y-27632 on relative mRNA expression levels of RhoA, ROCK1 and ROCK2, after cochlear excitotoxic injury. The relative mRNA expression levels were standardized by the expression levels before NK treatment in each mRNA (*n* = 6). *Indicates a significant difference (*p* < 0.05). The data are shown as the mean ± standard errors of mean.

**FIGURE 3:**
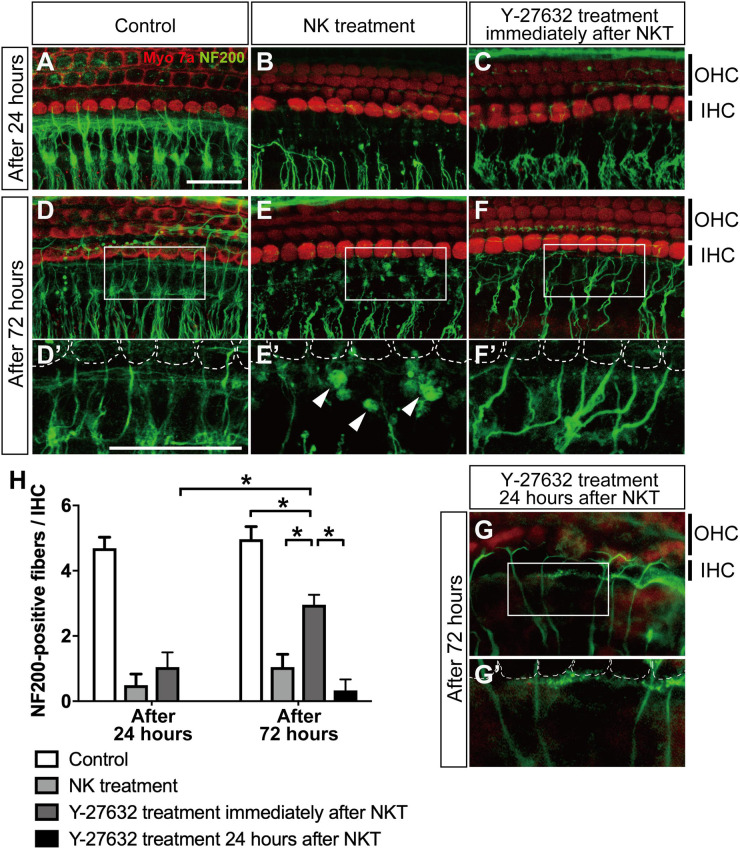
The effect of Y-27632 on the degenerated peripheral axons of the spiral ganglion after cochlear excitotoxic injury. In panels **(A–G)**, hair cells are labeled with anti-myosin 7a (red) and spiral ganglion peripheral processes with anti-NF200 (green). **(A–C)** z-Projections of confocal image stacks of the organ of Corti in explants fixed at 24 h after the end of NK exposure: without NK treatment (control) **(A)**, after NK treatment **(B)**, and with Y-27632 treatment **(C)**. **(D–F)** z-Projections of confocal image stacks of the organ of Corti in explants fixed at 24 h after the end of NK exposure: without NK treatment (control) **(D)**, after NK treatment **(E)**, and with Y-27632 treatment **(F)**. **(D′–F′)** Enlarged images of the white inlets in panels **(D,E)** focusing on the inner hair cells and the end of the peripheral axons of the spiral ganglion. White dotted lines show the contour of the inner hair cells. Arrowheads **(E′)** show the debris formed at the end of a peripheral axon. **(G,G′)** The peripheral ends of the axons in the SGN were elongated and some had connections with the IHC edge after Y-27632 treatment even at 24 h after NK treatment. Scale bar: 20 μm. **(H)** Counts of peripheral axons at two time points after NK treatment (24 and 72 h) with and without Y-27632, and 72-h treatment of Y-27632 even at 24 h after NK treatment (*n* = 6). *Indicates a significant difference (*p* < 0.05). The data are shown as the mean ± standard deviation.

Axonal growth was quantified as the average number of NF200-labeled fibers in contact with an IHC and was obtained by counting contacts in multiple confocal series, each of which contains 10–12 hair cells. This number was graphed as fibers/IHCs (Figures 1C, 2G). Presynaptic ribbons and postsynaptic densities were visualized as CtBP2 and PSD-95 immunoreactive puncta, respectively. Innervation was quantified by counting the number of postsynaptic densities on each IHC in z-stacks containing the bases of the IHCs. Individual postsynaptic densities in the stacks can be resolved and counted using an unbiased stereological method as previously reported (Wang and Green, 2011). The number of synapses made by each IHC was quantified by counting CtBP2 and PSD-95 puncta in contact with the IHC. This number was graphed (see Figure 3D) as CtBP2/IHC, or PSD-95 puncta/IHC. The survival rates of IHCs and OHCs, stained by myosin 7a, per field of view were also calculated in the same field by measuring the ratio of the total number of inner or outer hair cells and the number of inner or outer hair cells lost. Hair cells missing from the alignment were defined as lost. Observations were made within the range where the mean number of IHCs was 10–12.

### Statistical Analysis

The hair cell and synaptic count data were compared by one-way analysis of variance (*n* = 6 each group). Mean values were compared by one-way analysis of variance, followed by a *post hoc* multiple comparison procedure (Kruskal–Wallis and Dann-Bonferroni tests). The two-tailed Mann–Whitney *U* test was employed to compare differences in mRNA expression. Error bars represent the standard deviation of the mean. All statistical analyses were performed using Prism 7 (GraphPad Software Inc., La Jolla, CA, United States). *P*-values <0.05 were considered statistically significant.

## Results

### Excitotoxic Injury and ROCK1, ROCK2 Expression in the SGN and Peripheral Axons After Treatment With NK in an Organotypic Culture of the Cochlea

We first confirmed that the hair cells and peripheral axons in the SGN were morphologically damaged after 2 h of treatment with NK. The number of NF200-positive fibers/IHCs was significantly decreased immediately after treatment with NK (Figures 1A–C). The mean number of NF200-positive fibers/IHCs was 1.18 ± 0.77 after treatment with NK and 4.77 ± 0.34 in the control group at the same time point (Figures 1A–C). Meanwhile, the number of IHCs/OHCs did not change regardless of whether or not NK was administered (data not shown).

Local expression of ROCK1 and ROCK2 were confirmed by immunohistochemistry. Slight ROCK1 expression was identified in the cytoplasm of the somata in the SGN (Figures 1F–F″) but was not clearly identified in the peripheral axons (Figures 1D–D″). After excitotoxic damage by NK, debris-like expression of ROCK1 was observed around the peripheral ends of the SGN axons (Figures 1E–E″). These debris-like expression of ROCK1 was also observed around the somata of the SGN (Figures 1G–G″). NF200 allowed the identification of the constant expression of ROCK2 in the cytoplasm of the somata in the SGN (Figures 1J–J″) but the expression was not clearly identified in the peripheral axons (Figures 1H–H″). However, after excitotoxic damage by NK, the peripheral ends of the SGN axons showed marked degeneration and aggregation that had the appearance of debris formation (Figure 1I, white arrowhead). Expression of ROCK2 was significantly increased at the site of the debris (Figures 1I′,I″). In contrast, the morphology and ROCK2 expression pattern in the somata of the SGN did not change regardless of treatment with NK (Figures 1K–K″).

### Effect of Y-27632 on the mRNA Expression Level of RhoA, ROCK1, and ROCK2 in the Cochlear Tissue With Excitotoxic Injury

We confirmed the expression patterns of RhoA, ROCK1, and ROCK2 in the cultured cochlea before and after the excitotoxic damage caused by NK. In the intact cochlea, qPCR analysis revealed that mRNA expression levels of RhoA, ROCK1, and ROCK2 were increased (Figure 2). The mRNA expression levels of RhoA and ROCK1 were further increased even after 72-h culture (*p* < 0.0001, RhoA control vs 72 h after NK treatment; *p* = 0.0002, RhoA at 2 vs 72 h after NK treatment; *p* = 0.0284, ROCK1 control vs 72 h after NK treatment). Conversely, the mRNA expression level of RhoA significantly decreased in the Y-27632-treated group compared to the 72-h treatment of the NK group. The expression levels of ROCK1 and ROCK2 in the Y-27632-treated group decreased to near the expression level of the control group, whereas the differences in the 72-h treatment NK group were not significant.

### Effect of the ROCK Inhibitor Y-27632 on Neurite Growth in the Cochlea With Excitotoxic Injury

After we confirmed excitotoxic auditory nerve injury in the cultured cochlea, we applied Y-27632 to detect the regenerative effect in the damaged peripheral axon. Twenty-four hours after the excitotoxic injury induced by NK, the peripheral axons in the SGN were degenerated and the peripheral ends of the axons were still aggregated in the same way they had been in the same location immediately after treatment with NK (Figure 3B). The mean number of peripheral axons/IHCs in the SGN was 0.49 ± 0.34 in the NK group and 4.69 ± 0.33 in the control group (without NK; Figure 3A) with significant difference compared to the NK group (Figure 3H). At the same time point, explants with excitotoxic injury treated with Y-27632 also showed degeneration of the ends of the peripheral axons in the SGN; the mean number present was 1.05 ± 0.45/IHC, which was not significantly different from that in the NK group (Figures 3C,D).

Seventy-two hours after excitotoxic injury, degenerative changes and aggregation were still observed at the ends of the peripheral axons in the SGN (Figure 3E′, white arrowheads) in the group that was not treated with the ROCK inhibitor (Figure 3E), although the morphological structure in the same region remained in the control group (Figures 3D,D′). However, in the group treated with Y-27632 after excitotoxic injury at the same time point, the peripheral ends of the axons in the SGN were elongated and some had connections with the edge of the IHC (Figure 3F′, white dotted line); the number of peripheral axons/IHCs in the SGN was 2.96 ± 0.30, which was significantly greater than that at 24 h after treatment with Y-27632 (Figure 2H). This increase was also significant in comparison with the value in the NK treatment group at the same time point. However, the number of peripheral axons in the SGN 72 h after administration of the ROCK inhibitor following treatment with NK remained significantly decreased in comparison with the number in the intact explant at the same time point (Figure 3H). We also confirmed whether the elongation of axons was observed by treatment of Y-27632 even at 24 h after excitotoxic injury. The peripheral ends of the axons in the SGN were elongated and some had connections with the IHC edge in some samples (Figures 3G,G′), and there were many axonal spheroids at the end of the axons in the NK treatment group (Figure 3E) while axonal spheroids were mostly absent in the Y-27632 treatment 24 h after NKT group (Figure 3G); however, the mean number of peripheral axons/IHCs in the SGN was 0.40 ± 0.89, which was not significantly different from that in the NK group (Figure 3H).

Next, we examined the IHC and OHC survival rates at the same time points by counting the axons in all the study groups. There was no significant change in the IHC and OHC survival rates at any time point regardless of whether or not NK was administered. Furthermore, there was no significant change in the IHC and OHC survival rates after administration of Y-27632 in the group that was treated with NK (data not shown).

### Effect of Y-27632 on Acceleration of Synapse Formation in Cochlear Tissue With Excitotoxic Injury

**FIGURE 4:**
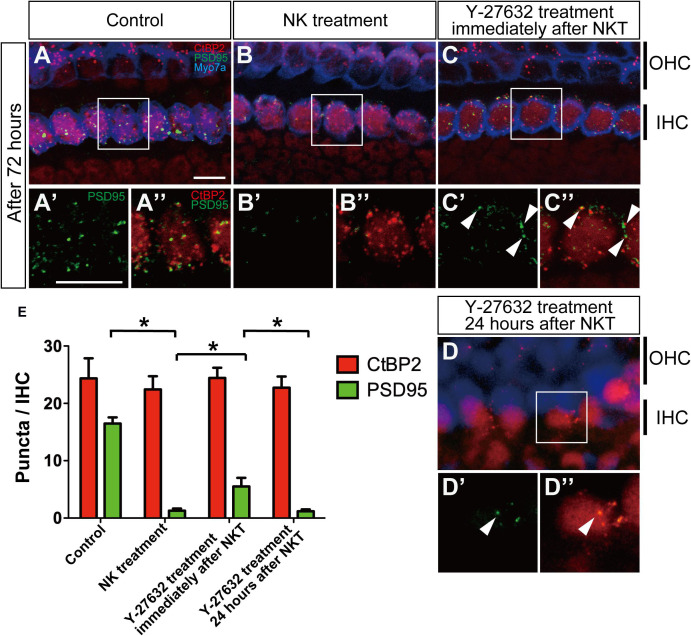
The effect of Y-27632 on the degenerated synapses of the inner hair cells 72 h after cochlear excitotoxic injury. In **(A–D),** presynaptic ribbons (CTBP2-immunoreactive puncta, red) and postsynaptic densities (PSD-95 immunoreactive puncta, green), and hair cells labeled with myosin 7a (blue) are shown. **(A′–D′,A″–D″)** Enlarged images of the white inlets in panels **(A–D)** focusing on the inner hair cells area. Panels **(A′–D′)** Show postsynaptic densities and double-labeled images with presynaptic ribbons are shown in panels **(A″–D″)**. White arrowheads in panel **(C″)** show typical overlapping synaptic density with CtBP2 and PSD-95 after Y-27632 treatment. **(A′–D′,A″–D″)** Scale bar: 20 μm. **(E)** Counts of puncta of presynaptic ribbons and postsynaptic densities 72 h after NK treatment with and without Y-27632, and 72-h Y-27632 treatment even at 24 h after NK treatment (*n* = 6). ^∗^Indicates a significant difference (*p* < 0.05). The data are shown as the mean ± standard deviation.

Finally, we quantified the number of synaptic markers in the IHCs, including presynaptic ribbons stained by CtBP2 and postsynaptic densities stained by PSD-95, to explore the functional connection between IHCs and the peripheral axons in the SGN at 72 h after administration of Y-27632. PSD-95 puncta in the NK-treated group were markedly decreased in comparison with the control group (Figures 4A,A′,B,B′). The mean number of PSD-95 puncta was 16.49 ± 1.10 in the control group and 1.30 ± 0.36 in the NK group (Figure 4E). However, the mean number of CtBP2 puncta was 24.53 ± 3.12 in the control group and 22.45 ± 2.28 in the NK group; the difference was not statistically significant and there were no morphological changes (Figures 4A,A″,B,B″). However, there was a significant increase in the mean number of PSD-95 puncta after administration of Y-27632 (5.52 ± 1.50) in comparison with the group that was not treated with Y-27632 (Figure 4E). Despite the significant increase in PSD-95 puncta in the Y-27632-treated group, the number in this group was still significantly lower than that in the control group (Figure 4E). The regenerated PSD-95 puncta were located beside the CtPB2 puncta around the IHC outline, which was considered to be the same region as the regenerated synapse (Figures 4C′,C″, arrowheads). Even in the Y-27632-treated group, there were no significant changes in the mean CtBP2 puncta count (24.45 ± 1.77; Figure 3E). Conversely, PSD-95 puncta in the treatment of Y-27632 even at 24 h after, decreased in the NKT group compared with the Y-27632-treated group (Figures 4D,D′) and CtBP2 puncta did not change (Figures 4D,D″). PSD-95 and CtBP2 puncta were 1.18 ± 0.38 and 20.15 ± 1.87, respectively (Figure 4E). There were not significantly different from PSD95 and CtBP2 puncta in the NK group.

## Discussion

In this study, we observed the regenerative effects of a ROCK inhibitor on NK-treated cochlear tissue, which represents excitotoxic damage, in terms of elongation of axons in the SGN and formation of synapses. We also found that mRNA expression levels of RhoA, ROCK1, and ROCK2 were increased in NK-treated cochlea. While expression of ROCK1 was higher in the whole cochlea, ROCK2 was expressed by the degenerated peripheral axons in the SGN of the NK-treated cochlea. The increased mRNA expression levels of the Rho-ROCK pathway may be suppressed by administration of Y-27632. The changes in the expression levels of RhoA, ROCK1, and ROCK2 and the localized expression of ROCK2 clearly show that the Rho/ROCK pathway is involved in the etiology of degeneration of the spiral ganglion and synapses and their regeneration. This is the first study to confirm that treatment with a ROCK inhibitor can regenerate SGN axons and synapses between IHCs and the auditory nerve in the cochlea after excitotoxic injury.

The mechanism by which of ROCK inhibitors cause regeneration of axons in the central nervous system has been investigated in detail. Axonal elongation requires reorganization of the actin cytoskeleton at the growth cone followed by orientation and stabilization of the microtubules. Activation of RhoA suppresses elongation of axons, and activation of Rac and Cdc42 has been shown to promote their elongation (Sarner et al., 2000; Yamaguchi et al., 2001). ROCK plays an important role in the suppression of axonal outgrowth downstream of RhoA (Govek et al., 2005), and it has been suggested that LIM kinase suppresses depolymerization of actin downstream of ROCK (Bito et al., 2000). Axonal regeneration after injury is due to physical damage from glial scars and myelin/oligodendrocyte-derived axon extension inhibitors, myelin-associated glycoproteins, Nogo-A, chondroitin sulfate proteoglycans (CSPGs), and oligodendrocyte myelin glycoprotein (Schwab, 2004). The effects of these inhibitors are suppressed by inactivation of RhoA and ROCK inhibitors (Alabed et al., 2006; Gopalakrishnan et al., 2008). ROCK2-deficient mice have been reported to recover after axonal injury (Duffy et al., 2009).

In contrast, peripheral axons are known to regenerate spontaneously, but functional reinnervation of the injured axon is often difficult (Navarro et al., 1997). Sensory, motor, and autonomic neurons, Schwann cells, other glial cells, and immune system cells were contained in the peripheral nervous system (Menorca et al., 2013). When peripheral nerves are injured, a process called Wallerian degeneration is initiated, which facilitates the removal of cell debris and enhances conditions favorable for axon regeneration (Rotshenker, 2011). At this time, Schwann cells form a structure called Büngner’s band. The Büngner’s band secures a scaffold for regenerating axons and supplies neurotrophic factors to help in the growth of regenerating axons (Webber and Zochodne, 2010). Schwann cells also have a phagocytic effect on myelin debris (Lyons et al., 2005), in contrast to glial cells, which suppress nerve regeneration by glial scar formation and persistence of myelin/oligodendrocyte-derived axon extension inhibitors in the central nervous system. However, it has been elucidated that the peripheral nervous system also contains inhibitory components of axon regeneration, such as CSPGs (Zuo et al., 1998a, b, c). Joshi et al. reported on the involvement of CSPGs in the peripheral motor neuron and the regenerative effect of ROCK inhibitors (Joshi et al., 2015). Although the peripheral sensory nerves did not regenerate in their study, they explained that the ability to regenerate depends on their responsiveness to CSPGs and ROCK inhibitors. In this study, we focused on the auditory nerve, which is one of the sensory nerves; however, the suppression of the mRNA expression levels of the Rho-ROCK pathway by Y-27632 administration was observed, as in the aforementioned study. These molecular biological changes suggested the possibility of regeneration of the sensory nerves when they present reactivity to CSPGs or ROCK inhibitors.

Studies on regeneration of the trigeminal nerve of zebrafish larvae indicate that NgR, its coreceptor leucine-rich repeat, immunoglobulin-like domain-containing protein 1 (LINGO-1), RhoA, ROCK, and their intracellular CRMP2 partners work as local inhibitor factors, while the ability of injured axons to reinnervate was improved by antagonizing these factors (O’Brien et al., 2009).

It is known that the tropomyosin receptor kinase (Trk) receptor has a high affinity for neurotrophin-3 (NT-3) and that p75 is a low affinity receptor (Kaplan and Miller, 2000; Reichardt, 2006). Trk C is expressed in the cochlea, and Wang and Green (2011) reported that inhibition of Trk C attenuated the nerve fiber regenerating effect of NT-3. Therefore, it is thought that the Trk C receptor pathway is involved in NT-3 activity in the inner ear. Furthermore, NT-3 has been reported to have a regenerating effect on auditory nerve fibers *in vivo* (Suzuki et al., 2016). In contrast, p75 has been implicated in the formation of dendrites downstream of brain-derived neurotrophic growth factor in the inner ear (Inoue et al., 2014) and is known to control axonal outgrowth in the central nervous system (Lee et al., 1994). p75 binds to and activates RhoA whereas binding of nerve growth factor to p75 has been shown to suppress RhoA activity and promote neurite outgrowth (Yamashita et al., 1999). p75 and Trk receptors are known to interact with each other (Numakawa et al., 2010; Gomes et al., 2012). The ROCK pathway is the downstream protein kinase of these neurotrophins, which include NT-3, nerve growth factor, and brain-derived neurotrophic growth factor (Yamashita et al., 1999; Kaplan and Miller, 2000). Therefore, ROCK inhibition is thought to be a relatively direct mechanism of axonal regeneration. Moreover, it would be easier to apply a ROCK inhibitor for neuronal regeneration in the clinical setting because such agents are already in clinical use. Our present findings point to the possibility of a breakthrough therapeutic strategy for hearing impairment accompanied by primary neural/synaptic degeneration.

In this study, morphological damage of hair cells was not observed in the cultured cochlear tissue. Therefore, we speculated that the ROCK inhibitor at the concentration used in this study had no or very limited toxicity to hair cells. There are few reports on the Rho/ROCK pathway in the cochlear region of the inner ear. With regard to hair cell damage, there are reports on activation of the ROCK pathway in OHCs that have been subjected to acoustic trauma (Chen et al., 2012) and on the protective effect of a Rac inhibitor against drug injury in OHCs (Bodmer et al., 2002). Those studies investigated the activity of the Rho/ROCK and Rac/Cdc42 pathways in OHC disorders. It has been shown that the activity of Rho decreases and conversely that the activity of Rac increases in OHCs that have sustained acoustic trauma or have been damaged by exposure to aminoglycoside antibiotic preparations. Chen et al. (2012) reported that inhibition of the activity of Rho strongly impaired OHCs while Bodmer et al. (2002) reported that inhibition of the activity of Rac had a protective effect on OHCs. These reports indicate that the balance between the Rho/ROCK and Rac/Cdc42 pathways determines the impairment of OHCs. An increase in the activity of the Rho/ROCK pathway or a decrease in the activity of the Rac/Cdc42 pathway is presumed to promote the survival of OHCs.

The main limitation of this research is that the results were obtained over a short observation period and a limited drug concentration. When Y-27632 was administered at 24 h after NK treatment, there was no significant change as a whole; however, some cases of axonal elongation could be confirmed. This result suggested that the reactivity may decrease depending on the time until drug administration. In addition, as the mRNA expression levels of RhoA were further increased at 72 h after NK treatment, it was suggested that the reactivity may have decreased due to insufficient drug concentration. Therefore, the implication of Y-27632 in the clinical situation would be at an earlier timepoint after hearing loss onset, similar to other preventive drugs. Future studies should include a wide range of observation periods and drug concentrations. Another limitation is that the results of this study only confirmed the effects on morphology but not the effects on hearing. Further research should include an analysis of the effect of ROCK inhibition on nerve elongation and formation of synapse in an *in vivo* model to elucidate the role of the Rho/ROCK and Rac/Cdc42 pathways in the cochlea.

In summary, this study has demonstrated that the Rho/ROCK pathway is involved in axon elongation in the cochlear spiral ganglion even after excitotoxic injury. The Rho/ROCK pathway was involved downstream of neurotrophins and their receptors in nerve regeneration in the cochlea. An advantage of the ROCK inhibitor used in this study is that it is already in clinical use. Further analysis of the RhoA/ROCK pathway in the process of regeneration of the cochlear spiral ganglion and the effect of a ROCK inhibitor in this etiology *in vivo* are expected in the future.

## Data Availability Statement

The raw data supporting the conclusions of this article will be made available by the authors, without undue reservation.

## Ethics Statement

The animal study was reviewed and approved by Yamagata University Animal Experiment Committee.

## Author Contributions

SK organized the whole study. YK, TI, KM, and SK were involved in conceptualization and design of the study. YK and TI conducted the research and analyzed the data. YK wrote early drafts of the manuscript. KM and SK prepared the manuscript. All authors reviewed the final version of the present manuscript.

## Conflict of Interest

The authors declare that the research was conducted in the absence of any commercial or financial relationships that could be construed as a potential conflict of interest.

## Footnotes

1 https://imagej.nih.gov/ij/
